# 3D Modeling of Self-Expandable Valves for PPVI in Distinct RVOT Morphologies

**DOI:** 10.1007/s00246-025-03796-7

**Published:** 2025-02-09

**Authors:** Ender Odemis, İbrahim Başar Aka, Mete Han Kızılkaya

**Affiliations:** 1 Faculty of Medicine, Department of Pediatric Cardiology, Koc University, Istanbul, Turkey; 2 Graduate School of Science and Engineering Department of Biomedical Science and Engineering, Koc University, Istanbul, Turkey; 3 Faculty of Engineering and Natural Sciences, Department of Mechatronics Engineering, İstanbul Bilgi University, Istanbul, Turkey

**Keywords:** Percutaneous pulmonary valve implantation, Right ventricular outflow tract, 3D printing, Pulsta THV®, Tetralogy of Fallot, Self-expandable valves, Mock circulatory system

## Abstract

Tetralogy of Fallot often requires transannular patch repair, leading to pulmonary insufficiency. Percutaneous pulmonary valve implantation (PPVI) with self-expandable valves offers a promising alternative, especially for enlarged right ventricular Queryoutflow tracts (RVOT). Five RVOT types identified in patients with Tetralogy of Fallot reflect anatomical variations due to disease and prior surgeries. This study assesses the Pulsta THV® valve’s in vitro hemodynamic performance across these RVOT morphologies using 3D-printed models. Five RVOT morphologies were recreated as 3D models from patient-specific imaging data. The Pulsta THV® valves, available in 28, 30, and 32 mm sizes, were evaluated using the ViVitro Pulse Duplicator System at three cardiac outputs (2, 3.5, and 5 L/min). Hemodynamic performance was assessed by measuring regurgitation rates and pressure gradients in the left and right pulmonary arteries. The Pulsta THV® performed optimally in RVOT Types 1 and 2, demonstrating lower regurgitation rates and pressure gradients, particularly with larger valve sizes. Conversely, RVOT Types 3 and 5 showed increased pressure gradients and hemodynamic variability, indicating less favorable outcomes. The results highlighted the critical role of precise anatomical compatibility, with larger valve sizes proving more effective in enlarged RVOT geometry. Valve sizes tailored to specific RVOT morphologies can enhance PPVI outcomes. Types 1 and 2 are ideal for PPVI, while Types 3 and 5 present challenges due to hemodynamic variability. This study supports 3D modeling and in vitro testing for pre-procedural planning to reduce complications, with future research exploring dynamic imaging and materials mimicking tissue properties.

## Introduction

Tetralogy of Fallot (TOF) is the most common cyanotic congenital heart disease. In addition, the right ventricular outflow tract is generally affected in approximately 20% of congenital heart diseases [1]. Over the past decades, surgical techniques and postoperative intensive care advancements have led to high success rates and significantly reduced mortality [2–4]. However, despite these achievements, long-term morbidity, mainly due to transannular patch (TAP) repair and severe pulmonary insufficiency (PI) beyond the second decade of life, remains a major clinical challenge. Historically, surgical pulmonary valve replacement was the standard treatment for severe PI, involving various conduits or biological valves. Regardless of the material, these valves often become dysfunctional over time, necessitating repeated surgical interventions. This cycle contributes to increased mortality and morbidity while diminishing the quality of life during long-term follow-up [5].

In response to these challenges, percutaneous pulmonary valve implantation (PPVI) emerged in the early 2000s as a promising alternative to traditional surgical valve replacement [6–9]. PPVI offers a less invasive option that can extend the functional lifespan of previously repaired RVOTs without needing open-heart surgery.

The diverse morphology, size, and biomechanical characteristics of the right ventricular outflow tract (RVOT) in TOF patients undergoing TAP repair present unique challenges [10]. Consequently, only a subset qualifies for PPVI. Recent advances in self-expandable valve technology have expanded the potential pool of candidates, allowing for the consideration of patients with enlarged RVOTs who were previously deemed ineligible. Among these innovative solutions, the Pulsta THV®, a self-expandable valve, has gained attention, though current clinical data remain limited and primarily focused on selective cases [11–14]. Our prior research demonstrated that in vitro testing of the Pulsta THV® using three-dimensional (3D) printed models could aid in optimizing PPVI by facilitating appropriate valve sizing and pre-procedural evaluation of hemodynamic performance [15]. However, no comprehensive data currently examine valve performance across different RVOT morphologies.

Schievano et al. conducted a seminal study using cardiac Magnetic Resonance Imaging (MRI) to classify RVOTs for PPVI suitability, identifying five distinct morphological types [16] Understanding variability in RVOT morphology is crucial for assessing procedural suitability and minimizing the risk of complications, such as device embolization [17]. In parallel with 3D printing in general medicine, it is used increasingly in congenital heart diseases [18]. Studies have indicated that 3D reconstructed RVOT models derived from cardiac MRI can help operators better to predict procedural outcomes and success rates [19]. In vitro simulations with 3D-printed models offer more detailed hemodynamic insights [20].

This study aims to evaluate the suitability of different RVOT morphologies for implantation of the self-expandable Pulsta THV® and assess its hemodynamic performance across these anatomical variations. The primary goal is to understand the potential outcomes of Pulsta THV® implantation in diverse RVOT types and determine how these morphological differences affect valve performance.

## Materials and Methods

### Patient-Specific RVOT 3D Model Generation

Computerized Tomography Angiography (CTA) scans were obtained using a multidetector CT scanner (Somatom Definition Flash, Siemens Healthineers, Forchheim, Germany) with 128 detector rows while the patients were supine. A prospective ECG-gated multiphase scanning technique was employed to capture images at the systolic phase, optimizing the images for regions of interest, such as the RVOT and pulmonary arteries. Scans were conducted at 80 kV with a dose range of 40–100 mAs, leveraging automatic exposure control to ensure optimal image quality while minimizing radiation exposure. For enhanced visualization, an intravenous injection of a nonionic contrast medium (370 mg I/ml) was administered at a dose of 1.5 ml/kg using the bolus tracking method to time the acquisition. This technique ensured clear delineation of anatomical structures and facilitated targeted visualization of the RVOT and pulmonary artery branches.

The images were reconstructed with a slice thickness of 0.75 mm, focused on the systolic phase of the cardiac cycle when the RVOT and pulmonary arteries exhibit maximum expansion. Extra precautions were taken to minimize motion artifacts from patient movement or breathing, maintaining high-resolution imaging quality. This process achieved high signal density and contrast levels suitable for generating precise 3D volumetric images of the RVOT, including detailed views of the main pulmonary artery and its branches.

Using Inobitech DICOM Viewer Pro®, 3D segmentation and modeling of the patient’s CTA images were performed, focusing on the RVOT, main pulmonary artery, and proximal branches. The segmentation isolated the relevant cardiovascular structures, converting DICOM data to Standard Triangle Language (STL) format to facilitate further processing for 3D printing. The segmented blood pool was subtracted from the anatomical wall structures to create anatomically accurate hollow models, resulting in a model ready for printing and further analysis.

### Blender for 3D Model Preparation

Blender, a versatile open-source 3D creation tool, prepared patient-specific RVOT models for 3D printing. The process began with importing the segmented and meshed models, saved in STL or OBJ formats, into Blender. Once imported, each model was carefully inspected for common mesh errors, such as non-manifold edges or intersecting faces, which could disrupt the 3D printing process. Blender’s “Edit Mode” and “Mesh Repair” tools were used to correct these errors, ensuring a clean and accurate base for further refinement.

To optimize the model geometry and reduce the polygon count, the “Decimate Modifier”was applied, which minimizes the file size and printing complexity without sacrificing anatomical accuracy. For models requiring hollowing to conserve printing material, the “Boolean Modifier” was utilized to create appropriate wall thicknesses, essential for mimicking accurate tissue dimensions.

**Fig. 1:**
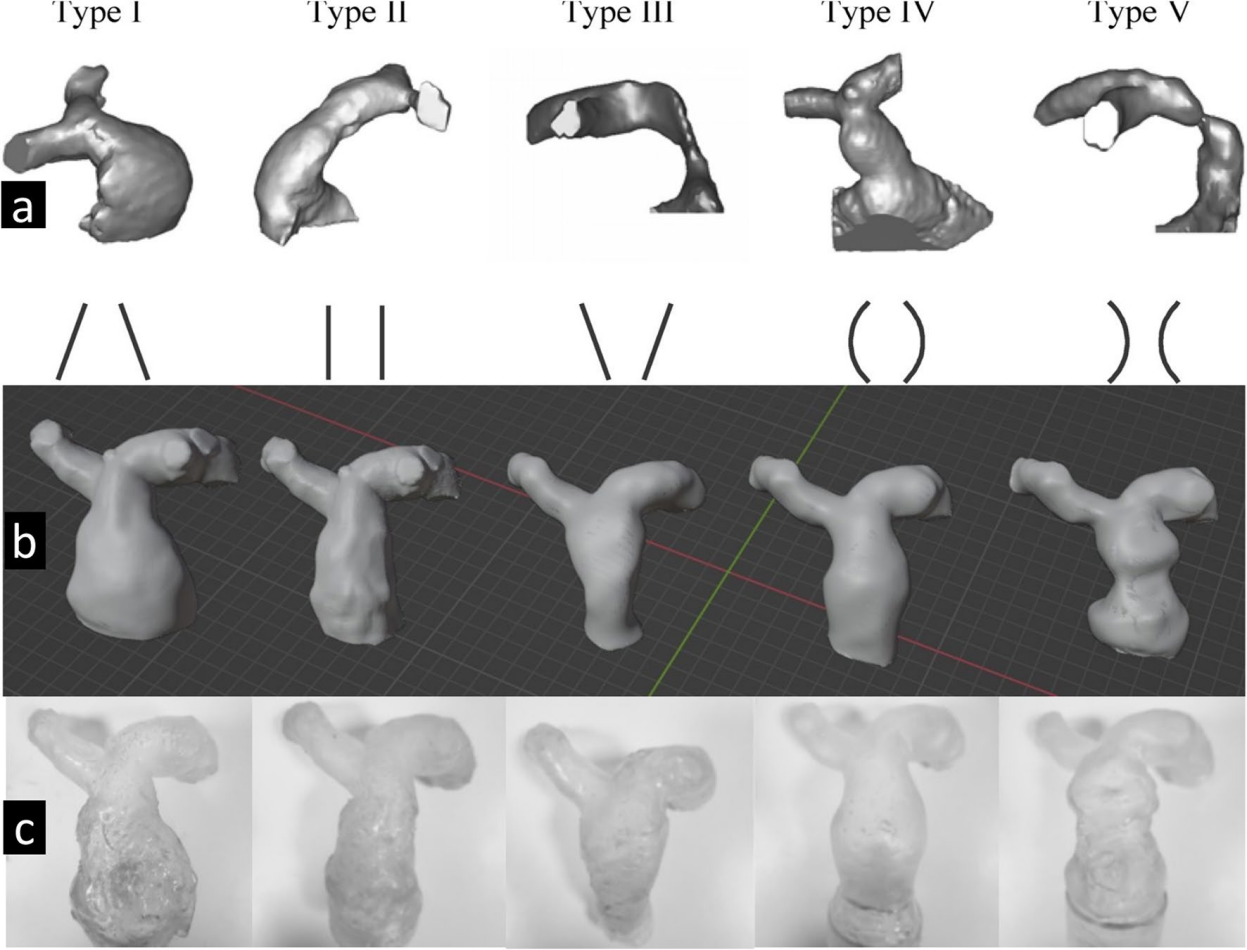
**a** Five distinct RVOT morphology (taken from Schievano et al.’s study). **b** RVOT Type 1 to Type 5 Fabricated Models. **c** 3D-Printed RVOT Types with 50A

In this study, RVOT models of five types were developed based on an original patient-specific model previously identified as RVOT Type 2. Following existing literature and anatomical guidelines, we created additional RVOT configurations (Types 1, 3, 4, and 5) by modifying the Type 2 model in Blender to match the dimensional and structural characteristics of each RVOT type (Fig. 1A). All RVOT models were designed to maintain consistency with a primary pulmonary artery diameter of 27 mm in the middle segment (Fig. 1B). This standardization allowed for direct comparison across types, ensuring anatomical precision and readiness for 3D printing.

The studies involving humans were approved by the Koç University Faculty of Medicine Ethical Committee. The studies were conducted according to local legislation and institutional requirements. The national legislation and institutional requirements did not require written informed consent for participation from the participants or the participants’ legal guardians/next of kin.

### Fabrication of the Pulmonary Artery Model

High-resolution resin 3D printers, specifically Formlabs Form3 printers, were selected for model fabrication due to their ability to produce fine anatomical details. Each RVOT-type model was printed using 50A resin, chosen for its biocompatibility and mechanical properties, which closely resemble those of human vascular tissue. The model was subjected to fluoroscopic imaging to simulate transcatheter valve placement under realistic conditions, mirroring real-world procedural conditions.

Key-printing parameters, such as layer height and support structures, were meticulously configured to ensure optimal print quality. After printing, the models underwent post-processing steps, including rinsing in isopropyl alcohol, UV curing to solidify the resin, and carefully removing support structures. These steps produced a high-fidelity pulmonary artery model that accurately replicated anatomical features, making it suitable for applications in surgical planning and device testing (Fig. 1C).

For PPVI procedures, the appropriate valve diameter is typically determined to be approximately 10% or 2–4 mm larger than the main pulmonary artery (MPA) implantation area size [21]. Given the 27 mm diameter of the main pulmonary artery in our models, the optimal valve diameter was set at 30 mm for the landing zone. This study also aimed to examine the hemodynamic effects of valve sizes slightly above and below this optimal measurement; thus, valve diameters of 28 mm and 32 mm were selected to provide insights into configurations 2 mm smaller and 2 mm larger than the estimated valve diameter.

### Cardiovascular Mock Loop and Hemodynamic Measurements

Pulsatile flow testing is considered the gold standard for hemodynamic evaluation of prosthetic valves. The International Organization for Standardization (ISO) has established several hemodynamic standards to ensure consistency across laboratories and research studies. Nominal test conditions include a cardiac output (CO) of 5 L/min, a heart rate of 70 beats/min, and a systolic period comprising 35% of the cardiac cycle [22]

**Fig. 2:**
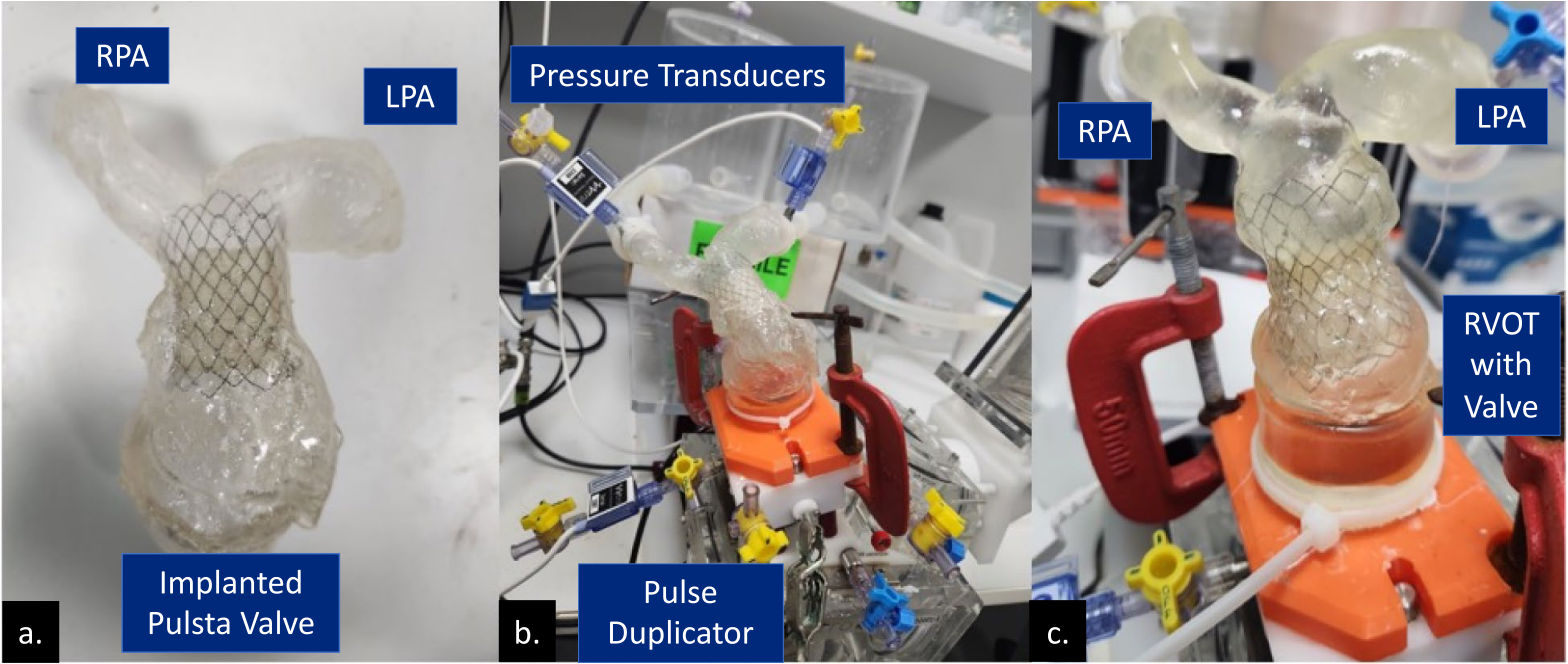
**a** Implanted Pulsta valve within a 3D-printed RVOT Type 1 model. **b** Experimental setup showing the RVOT Type 1 model equipped with a 32 mm valve attached to the ventricular outlet of the ViVitro Pulse Duplicator System (ViVitro Labs Inc., Victoria, BC) with connected pressure transducers for data acquisition. **c** Close-up view of the RVOT Type 5 model featuring a 28 mm valve. (*RPA* right pulmonary artery, *LPA* Left pulmonary artery, *RVOT* Right ventricular outflow tract)

For this study, valves were tested using the ViVitro Pulse Duplicator System (ViVitro Labs Inc., Victoria, BC), a system designed to simulate physiological conditions as used in previous studies [20, 21, 23]. The system was configured to a systole-to-diastole ratio of 60/40, maintaining a mean arterial pressure (MAP) of 20 mmHg. While physiological CO ranges from 4 to 5 L/min, this study examined valve performance at three flow rates—2, 3.5, and 5 L/min—to replicate various physiological conditions and assess valve performance over a range of cardiac outputs. Testing was conducted at a heart rate of 80 beats/min (Fig. 2). Using normotensive pressure values enhanced the evaluation of valve insufficiency by minimizing the influence of simulated pressures on the testing conditions [21]. To maintain hemodynamic consistency, distilled water at room temperature, matched to blood osmolarity, was used as the test fluid.

Regurgitation fractions (RF) were measured using an electromagnetic flowmeter integrated into the ViVitro Pulse Duplicator System. Data were collected from 10 steady-state pulses to ensure accuracy and repeatability. This setup provided comprehensive data on regurgitation rates and pressure gradients within the Left Pulmonary Artery (LPA) and Right Pulmonary Artery (RPA) across different flow rates and valve sizes, facilitating a robust comparison of hemodynamic performance.

## Results

To assess hemodynamic performance across various configurations, three sizes (28, 30, and 32 mm) of the Pulsta THV® were tested across five RVOT configurations under three cardiac outputs (2, 3.5, and 5 L/min). This setup produced nine combinations of RVOT type, valve size, and cardiac output, each tested in five replicates. Types 1, 2, and 4 underwent 45 experiments each, while types 3 and 5, which could not accommodate the 32 mm valve, had 30 experiments each. In total, 195 experiments were conducted, measuring key parameters such as regurgitation flow rates and pressure gradients in the LPA and RPA. Tables 1 and 2 summarize these findings.

**Table 1:**
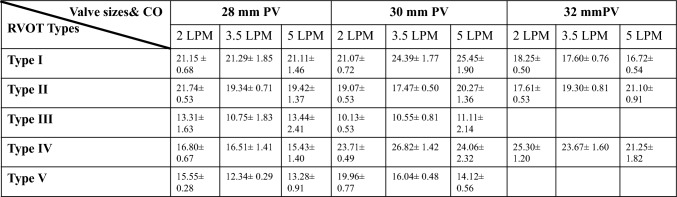
Summary of pulmonary regurgitation measurements across five different RVOT types, recorded at cardiac output levels of 2, 3.5, and 5 LPM with varying valve sizes

The table presents the regurgitation percentage (Reg %) and corresponding standard deviation (Std) for each experimental condition

**Table 2:** Gradients and standard deviations measured in LPA and RPA in experiments with 5 different RVOT types are shown in the table

RVOT Type	LPA Gradient (Mean ± SD)	RPA Gradient (Mean ± SD)
Type I	8.32 ± 5.12 mmHg	10.68 ± 5.50 mmHg
Type II	8.96 ± 4.62 mmHg	10.58 ± 5.50 mmHg
Type III	12.77 ± 9.11 mmHg	20.26 ± 11.80 mmHg
Type IV	11.32 ± 5.67 mmHg	14.00 ± 6.10 mmHg
Type V	11.43 ± 7.58 mmHg	23.14 ± 10.79 mmHg

### Regurgitation Flow Rates

RVOT Type 1 demonstrated the lowest overall regurgitation rates among all tested configurations. A minimum regurgitation of 16.72% (mean Std of 0.54 ± 0.1) was achieved with a 32 mm valve at a flow rate of 5 L/min. The regurgitation rate increased to approximately 25% (mean Std of 1.8 ± 0.1) with a 30 mm valve at flow rates of 3.5 and 5 L/min, suggesting heightened regurgitation at higher cardiac outputs. Experiments with 28 mm, 30 mm, and 32 mm valves at 5 Lpm CO showed 20.35%, 26.25%, and 16.83%, respectively (Fig. 2).

In experiments conducted with 28 mm, 30 mm, and 32 mm valves at 5 LPM cardiac output for RVOT type 2, the regurgitation rate was measured as 19.42, 20.27, and 21.10, respectively, and similar results were obtained. RVOT Type 2 also exhibited favorable regurgitation performance, with the lowest rate at 17.5% (mean Std of 0.5 ± 0.03) observed using a 30 mm valve at a flow rate of 3.5 L/min.

Due to design constraints, only the 28 mm and 30 mm valves were tested for RVOT Type 3. The minimum regurgitation was observed at 10.1% (mean Std of 0.53 ± 0.05) with a 30 mm valve at a flow rate of 2 L/min. The maximum regurgitation, reaching 13.4% (mean Std of 2.41 ± 0.1), was observed with a 28 mm valve consistently across both 2 and 5 L/min flow rates, showing minimal variability in regurgitation rates.

In RVOT type 4, the regurgitation rates at 2, 3.5, and 5 L/min with the 28 mm valve were 16.80%, 16.51%, and 15.43%, respectively, much lower than those observed with the 30 mm and 32 mm valves. RVOT Type 4 achieved a minimum regurgitation rate of 15.43% (mean Std of 1.4 ± 0.05) with a 28 mm valve at 5 L/min, while approximately 24.06% (Std of 2.32 ± 0.1), occurred with a 30 mm valve at 5 L/min.

RVOT Type 5, despite not being tested with the 32 mm valve, exhibited the highest baseline regurgitation rates among the configurations. Minimum regurgitation was observed at 12.3% (mean Std of 0.296 ± 0.03) with a 28 mm valve at 2 L/min, while maximum regurgitation was recorded at 19.9% (mean Std of 0.771 ± 0.05) with a 30 mm valve at 2 L/min. This baseline level of regurgitation indicates that RVOT Type 5 may have limited suitability for scenarios requiring minimal regurgitation.

### Pressure Gradient Performance

RVOT Type 1 exhibited the most favorable pressure gradient performance, with mean LPA and RPA gradients of 8.32 mmHg and 10.68 mmHg, respectively. These values remained stable across all tested flow rates, positioning Type 1 as one of the most favorable configurations. The 32 mm valve performed exceptionally well, especially at lower flow rates, reinforcing Type 1’s overall stability in gradient performance. In experiments conducted with 28 mm, 30 mm, and 32 mm valves at 5 Lpm CO, the stenosis gradient was measured as 67.09 mmHg, 78.06 mmHg, and 63.17 mmHg, respectively.

RVOT Type 2 also showed favorable pressure gradients, closely comparable to Type 1. A mean LPA gradient of 8.96 mmHg and an RPA gradient of 10.58 mmHg were observed, with the lowest RPA gradient favoring efficient pulmonary flow. However, gradients increased at higher flow rates, with values such as 12.93 mmHg for LPA and 15.87 mmHg for RPA at 5 L/min, indicating some limitations at higher flow conditions.

In contrast, RVOT Type 3 displayed higher variability in gradient performance. A mean LPA gradient of 12.77 mmHg and an RPA gradient of 20.26 mmHg were recorded, with standard deviations indicating inconsistent flow behavior, especially under high-flow conditions. The larger gradients and significant variability, particularly at higher flow rates, reduce the favorability of Type 3 for consistent flow control.

RVOT Type 4 performed best at moderate flow rates, with mean LPA and RPA gradients of 11.32 mmHg and 14.00 mmHg, respectively. Although it displayed a more balanced performance than Type 3, it did not surpass Types 1 and 2 under lower flow conditions.

Finally, RVOT Type 5 exhibited the highest pressure gradients overall, particularly in the RPA, with a mean of 23.14 mmHg and a standard deviation of 10.79 mmHg. Due to its elevated gradient values, this configuration remained consistent under high-flow conditions but needed to be more suitable for scenarios where low-resistance flow is desired.

**Fig. 3:**
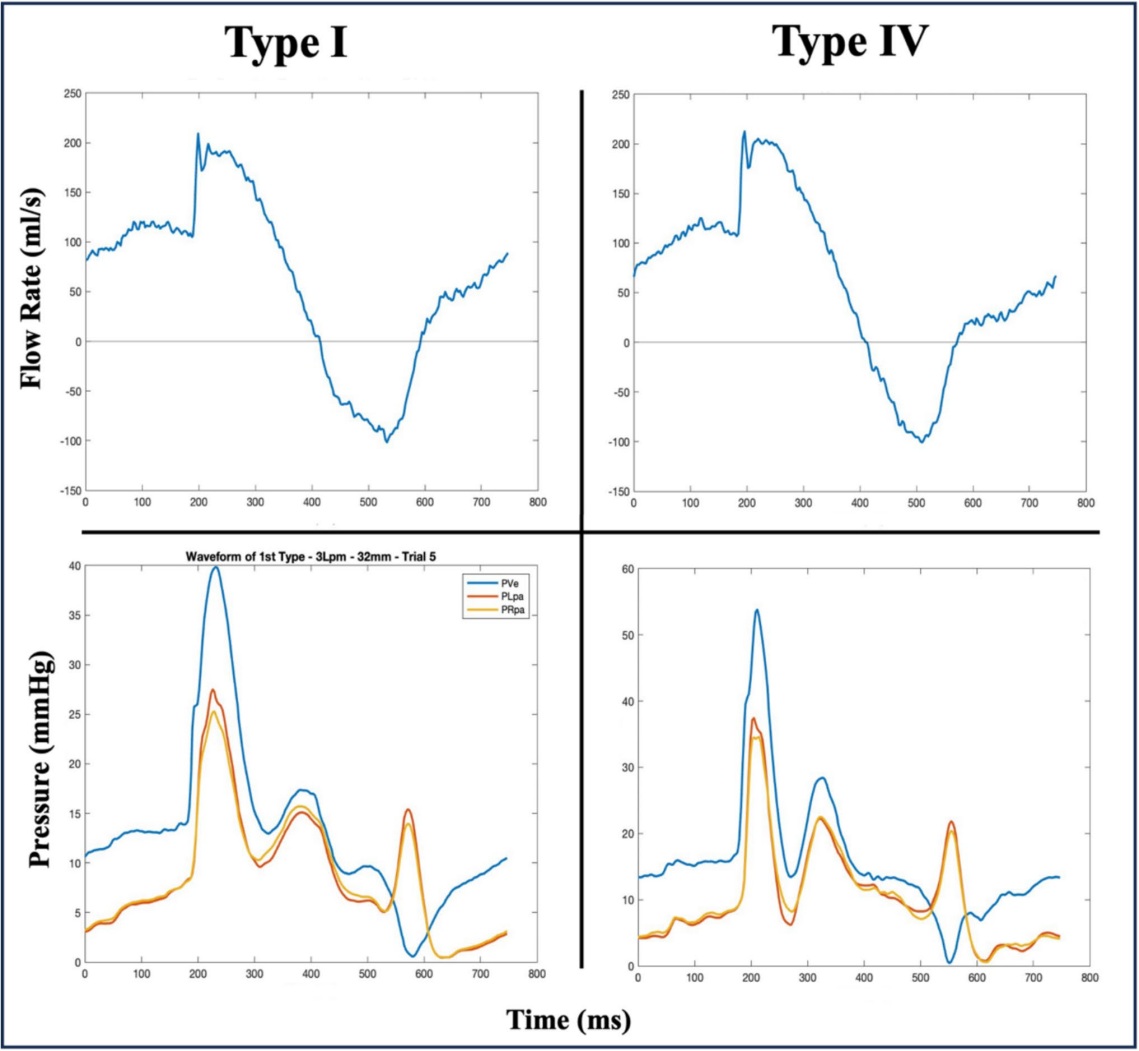
Comparative analysis of flow rate (top row) and pressure waveforms (bottom row) for two RVOT morphologies (Type 1 and Type 4), recorded at a constant flow rate of 3.5 LPM. The flow rate plots illustrate the PI flow characteristics specific to each RVOT type, while the pressure waveforms highlight the LPA and RPA flow gradients under identical conditions

Figure 3 illustrates the performance characteristics of Type 1 and Type 4 RVOT at 3.5 L/min.

## Discussion

Percutaneous pulmonary valve implantation (PPVI) has proven effective in treating severe pulmonary insufficiency (PI). However, significant anatomical variability exists in the right ventricular outflow tract (RVOT) among patients with congenital heart disease who have undergone surgical repairs, such as the Tetralogy of Fallot. These variations—including an enlarged RVOT, stenotic segments, and angles at the pulmonary artery bifurcation—impact the success of PPVI procedures and influence hemodynamic outcomes. To improve procedural success and reduce complications, it is crucial to establish a reliable landing zone and select the correct valve size using CT, MRI, and balloon-sizing methods [24]. The architecture of the RVOT displays intricate variability shaped by the underlying pathology and prior surgical intervention. Schievano et al. categorized RVOT morphologies into five types frequently referenced in clinical practice: Type 1 pyramidal, Type 2 straight, Type 3 inverted pyramidal, Type 4 fusiform, and Type 5 concave [25]. In this study, we employed a novel pulse duplicator system to analyze the hemodynamic performance of the Pulsta THV® across these RVOT types. Using the ViVitro Pulse Duplicator System (ViVitro Labs Inc., Victoria, BC), we evaluated different valve sizes (28, 30, and 32 mm) under varied cardiac outputs (2, 3.5, and 5 L/min), measuring PI and flow patterns in the LPA and RPA.

RVOT Type 1 consists of patients with significantly enlarged RVOTs, commonly following transannular patch (TAP) repair. Initially, PPVI in Type 1 RVOT patients using balloon-expandable valves was challenging due to the limited valve dimensions [16, 26]. Our experiments demonstrated that the larger valve size consistently had lower regurgitation rates across all cardiac outputs. These findings suggest that larger valves exceeding the predicted size offer superior outcomes in Type 1 RVOTs. Technological advancements have enabled PPVI with self-expandable systems that can accommodate larger RVOTs, aligning with our findings of improved outcomes in enlarged RVOTs when using larger valves. Sivaprakasam et al. reported successful PPVI in patients with large RVOTs by selecting valves 23–25% wider than balloon-sizing measurements, demonstrating that this oversizing approach, despite risks like pulmonary artery rupture or coronary compression, could ensure successful implantation in challenging anatomies [27]. Furthermore, the PT Valve (Beijing Med-Zenith, Beijing, China) has succeeded in pyramidal RVOTs. In two patients with large, pyramidal RVOTs, pre-procedural 3D models confirmed that implanting larger valves than those indicated by measurements could achieve favorable outcomes [28].

The more straightforward structure of Type 2 RVOT allows PPVI in the mid-pulmonary artery, and our study shows that selecting a valve size matching the measurements improves outcomes. Our results align with other studies indicating that Types 1 and 2 are the most favorable configurations for minimizing pressure gradients, especially under low to moderate flow rates. Both types demonstrated low mean gradients and low variability, making them suitable for clinical applications where minimal flow resistance is essential. For Type 2 anatomy, similar-sized valves to the measured dimensions typically demonstrated the lowest regurgitation rates, with optimal results at 3.5 L/min.

For Type 3 anatomy, both smaller and similar-sized valves showed low regurgitation rates, with the lowest regurgitation occurring at lower to moderate cardiac outputs (2 and 3.5 L/min) using a similar-sized valve. This indicates that a standard-sized valve is preferable for Type 3 patients at these flow rates. However, stabilizing the valve in Type 3 anatomy remains challenging, similar to issues encountered with Type 1. Park et al. indicated that larger valve sizes were often necessary for native RVOT configurations to address stability concerns, particularly in Types 1 and 3 [11]

In Type 4, our experiments demonstrated that regurgitation rates decreased across all cardiac output levels when smaller valves were used. However, due to stenotic regions at the proximal and distal ends of the RVOT and a broader area in the center, selecting larger valves early in the procedure may help prevent paravalvular leakage and improve RVOT coverage [25].

Type 5 anatomy benefited most from smaller valve sizes, achieving lower regurgitation rates at all cardiac output levels, suggesting that smaller valve sizes are more suitable for Type 5. However, Types 3 and 5 exhibited the highest pressure gradients and variability, indicating suboptimal performance for stable hemodynamics. The elevated gradients observed in these configurations suggest greater resistance, which may lead to unfavorable outcomes, particularly in high-flow scenarios. *Type 5 demonstrated consistent performance under high-flow conditions; however, the significantly elevated pressure gradients observed across the pulmonary arteries limit its effectiveness in applications requiring low-resistance flow.* In Type 5, the central region’s stenosis further complicates valve selection, and Park et al. emphasize a preference for smaller valves in this RVOT type to manage hemodynamic performance [11].

The role of 3D cardiac modeling and in vitro simulations in refining PPVI procedures is growing, providing a clearer spatial understanding of the anatomical relationships between the arteries and ventricles. These models allow for more precise surgical planning and adjustments to procedural techniques. For example, 3D-printed models have been used to simulate self-expandable valve implantation, allowing clinicians to test and adjust strategies before actual procedures [10]. Our results indicate that mock circulatory testing of valve sizes can optimize pre-procedural planning, helping clinicians select the appropriate valve size to avoid complications such as coronary artery compression. Moreover, the in vitro approach delivers reproducible, reliable data beyond imaging alone. Given that the compact design of the Pulsta THV® can lead to valve embolization if an inappropriate landing mark or under-sizing occurs, pre-procedural testing in 3D-printed models may reduce these risks [11]. Conversely, oversizing could potentially impair valve functionality. Thus, 3D printing and in vitro assessments support morphological evaluation and patient-specific valve sizing. *This study provides valuable insights into valve performance across different RVOT morphologies. However, the stiffer nature of the materials used in the *in vitro* model may not fully replicate the dynamic, compliant conditions observed in clinical settings. Future studies should aim to establish clinical correlations by integrating patient-specific CT scans to develop more physiologically representative models and comparing *in vitro* findings with actual valve function post-implantation.*

*To ensure the clinical utility of *in vitro* models for valve sizing and selection, it is essential to validate their findings through clinical correlation. This requires comparing outcomes from patient-specific CT-based models with actual post-implantation valve performance. Bridging this gap between experimental observations and clinical applications will enhance the translational value of these models.*

### Study Limitations

The 3D models were created based on systolic CT angiography images, which capture only a single phase of the cardiac cycle, potentially omitting dynamic changes that occur throughout. In addition, our mock system does not replicate surrounding tissue effects on valve function or assess long-term durability. Using silicone models that more closely mimic natural tissue properties could provide more accurate results, as the 50A Resin used for our models is notably stiffer than actual cardiac tissue.

## Conclusion

This study underscores the potential utility of the Pulsta THV® valve for PPVI across diverse RVOT anatomies and emphasizes the importance of selecting valve sizes tailored to each RVOT type. Our findings indicate that Type 1 and Type 2 configurations are particularly favorable for clinical application, especially when using larger valve sizes, as these configurations effectively reduce pressure gradients. Such configurations are well suited for patients undergoing PPVI, where minimizing flow resistance and maintaining balanced pulmonary circulation are essential for long-term success. In contrast, the increased gradients and variability observed in Types 3 and 5 suggest limitations for these anatomies, especially in patients with elevated cardiac output demands or high pulmonary pressures, as these factors could impact hemodynamic stability and overall valve performance.

Future research should focus on enhancing patient-specific modeling using more physiologically representative materials, such as silicone, to capture the dynamic characteristics of cardiac tissues better. This approach will improve pre-procedural planning, allowing clinicians to refine valve sizing and placement strategies, ultimately contributing to optimized long-term outcomes for PPVI patients.

### Clinical Perspectives

#### Competency in Medical Knowledge

This study demonstrates the importance of precise valve sizing in percutaneous pulmonary valve implantation (PPVI) for patients with diverse right ventricular outflow tract (RVOT) morphologies. In particular, RVOT Types 1 and 2 showed the most favorable hemodynamic performance when using the self-expandable Pulsta THV® valves. Types 3 and 5, however, exhibited increased gradients and regurgitation rates, indicating a need for precise anatomical assessment and careful valve selection to avoid suboptimal outcomes.

#### Competency in Patient Care

By employing 3D-printed models of patient-specific RVOT anatomies, this research provides a novel tool for pre-procedural planning in PPVI. Clinicians can leverage this approach to optimize valve sizing and positioning based on individual RVOT morphologies, which could minimize procedure-related complications and improve long-term outcomes.

#### Translational Outlook 

Future research should focus on enhancing the predictive accuracy of in vitro assessments by incorporating dynamic imaging modalities and biomimetic materials into 3D model fabrication. In addition, using larger patient datasets with varied RVOT anatomies may support the design of custom valve options that optimize hemodynamic performance across different anatomical configurations.

## Data Availability

No datasets were generated or analysed during the current study.

## Abbreviations

CTA: Computerized tomography angiography
MPA: Main pulmonary artery
RPA: Right pulmonary artery
LPA: Left pulmonary artery
PPVI: Percutaneous pulmonary valve implantation
RVOT: Right ventricular outflow tract
STL: Standard triangle language
TAP: Transannular patch
THV: Transcatheter heart valve
TOF: Tetralogy of fallot
